# Non-invasive measurement of tumor immune microenvironment and prediction of survival and chemotherapeutic benefits from ^18^F fluorodeoxyglucose PET/CT images in gastric cancer

**DOI:** 10.3389/fimmu.2022.1019386

**Published:** 2022-10-13

**Authors:** Junmeng Li, Chao Zhang, Huihui Guo, Shuang Li, Yang You, Peiming Zheng, Hongquan Zhang, Huanan Wang, Junwei Bai

**Affiliations:** ^1^ Department of Gastrointestinal Surgery, Henan Provincial People’s Hospital, Zhengzhou University People’s Hospital, Henan University People’s Hospital, Zhengzhou, China; ^2^ Department of Radiology, Henan Provincial People’s Hospital, Zhengzhou University People’s Hospital, Henan University People’s Hospital, Zhengzhou, Henan, China; ^3^ Department of Pathology, Henan Provincial People’s Hospital, Zhengzhou University People’s Hospital, Henan University People’s Hospital, Zhengzhou, China; ^4^ Department of Nuclear Medicine, Henan Provincial People’s Hospital, Zhengzhou University People’s Hospital, Henan University People’s Hospital, Zhengzhou, Henan, China; ^5^ Department of Clinical Laboratory, Henan Provincial People’s Hospital, Zhengzhou University People’s Hospital, Henan University People’s Hospital, Zhengzhou, China; ^6^ Department of Thoracic Surgery, The First Hospital Affiliated of Xinxiang Medical University, Xinxiang, China; ^7^ Department of Gastrointestinal Surgery, The First Hospital Affiliated of Zhengzhou University, Zhengzhou, China

**Keywords:** Gastric cancer, tumor immune microenvironment, chemotherapy, predictive, Positron Emission Tomography-Computed Tomography (PET/CT), radiomics

## Abstract

**Background:**

The tumor immune microenvironment could provide prognostic and predictive information. It is necessary to develop a noninvasive radiomics-based biomarker of a previously validated tumor immune microenvironment signature of gastric cancer (GC) with immunohistochemistry staining.

**Methods:**

A total of 230 patients (training (n = 153) or validation (n = 77) cohort) with gastric cancer were subjected to (Positron Emission Tomography-Computed Tomography) radiomics feature extraction (80 features). A radiomics tumor immune microenvironment score (RTIMS) was developed to predict the tumor immune microenvironment signature with LASSO logistic regression. Furthermore, we evaluated its relation with prognosis and chemotherapy benefits.

**Results:**

A 8-feature radiomics signature was established and validated (area under the curve=0.692 and 0.713). The RTIMS signature was significantly associated with disease-free survival and overall survival both in the training and validation cohort (all *P*<0.001). RTIMS was an independent prognostic factor in the Multivariate analysis. Further analysis revealed that high RTIMS patients benefitted from adjuvant chemotherapy (for DFS, stage II: HR 0.208(95% CI 0.061-0.711), p=0.012; stage III: HR 0.321(0.180-0.570), p<0.001, respectively); while there were no benefits from chemotherapy in a low RTIMS patients.

**Conclusion:**

This PET/CT radiomics model provided a promising way to assess the tumor immune microenvironment and to predict clinical outcomes and chemotherapy response. The RTIMS signature could be useful in estimating tumor immune microenvironment and predicting survival and chemotherapy benefit for patients with gastric cancer, when validated by further prospective randomized trials.

## Introduction

Extensive studies have suggested tumor immune microenvironment are of clinical importance in cancer progression, metastasis, therapeutic response (1–4). The type, density, and location of immune cells in multiple cancers had a prognostic value that was superior to and independent of those of the TNM stage (1, 5, 6). An international consortium of 14 centers in 13 countries assessed the Immunoscore assay in patients with TNM stage I-III colon cancer, and the results supported the implementation of the consensus Immunoscore as a new component of a TNM-Immune classification of cancer (1). For gastric cancer (GC), a tumor immune microenvironment (TME) signature (7) of gastric cancer based on seven features, including CD3 _invasive margin (IM)_, CD8 _IM_, CD45RO _center of tumor (CT)_, CD66b _IM_, CD34, periostin, and cyclooxygenase-2, which could be important for predicting survival and selecting appropriate patients for chemotherapy. However, the TME signature was mainly determined on postoperative tissue specimens, it is necessary to develop a noninvasive pretreatment tools for prediction of immune infiltrates.

Computational medical imaging, known as radiomics, is an emerging field that converts medical images into a high dimensional quantitative feature space using a large number of automatically extracted data-characterization algorithms (8–10). These imaging features may capture in-depth characterization of tumor distinct phenotypes, with the underlying hypothesis that imaging reflects not only macroscopic but also the cellular and molecular properties of tissues. The goal of radiomics is to develop image-driven biomarkers that serve as instruments that generate a further understanding of cancer biology to facilitate better clinical decision-making (10–12). Radiomics features are complementary to biopsies and have the advantages of being non-invasive and repeated during treatment in routine practice, contrary to genomics or proteomics, which are still challenging to apply in clinical routine (12, 13). Connecting radiomics features to the molecular biological processes active in a tumor could provide deeper information that may complement the molecular data (14). Hence, in some circumstances, the radiomics features could be apply to infer the molecular biological underpinnings of tumor in individual patients.

The association between imaging features and tumor infiltrating immune cell density has been explored (13, 15–17). Many image features extracted by radiomics, not visually observed, were closely related to specific microscopic features at the molecular level and could characterize the tumor and its tumor microenvironment (13, 18–20). Several studies also found that pre-existing tumor immune infiltration correlates with patient response to anti-programmed cell death protein (PD)-1 and anti-programmed cell death ligand 1 (PD-L1) immunotherapy (21, 22).

Gastric cancer is one of the most common cancer and leading cause of cancer death worldwide (23). Imaging with fluorine 18 (18F) fluorodeoxyglucose (FDG) positron emission tomography (PET) was used to detect of distant and lymph nodes metastases, which could stage GC accurately (24). PET/CT could reflect the tumor metabolically active. Chemotherapy have improved survival of GC patients (25, 26). However, for most of GC patients the survival rates were still limited despite initial high response rates (26, 27). Therefore, precise classification of GC that could be necessary to predict patient survival and chemotherapy responses. Radiomics could allow evaluation of a tumor and its microenvironment, thus, may lead to the identification of novel predictors for prognosis and treatment efficiency. We aimed to develop a PET/CT image based radiomics signature of tumor immune microenvironment and to assess the ability of this signature to predict survival and adjuvant chemotherapy benefits.

## Patients and methods

### Study design and patients

**Figure 1:**
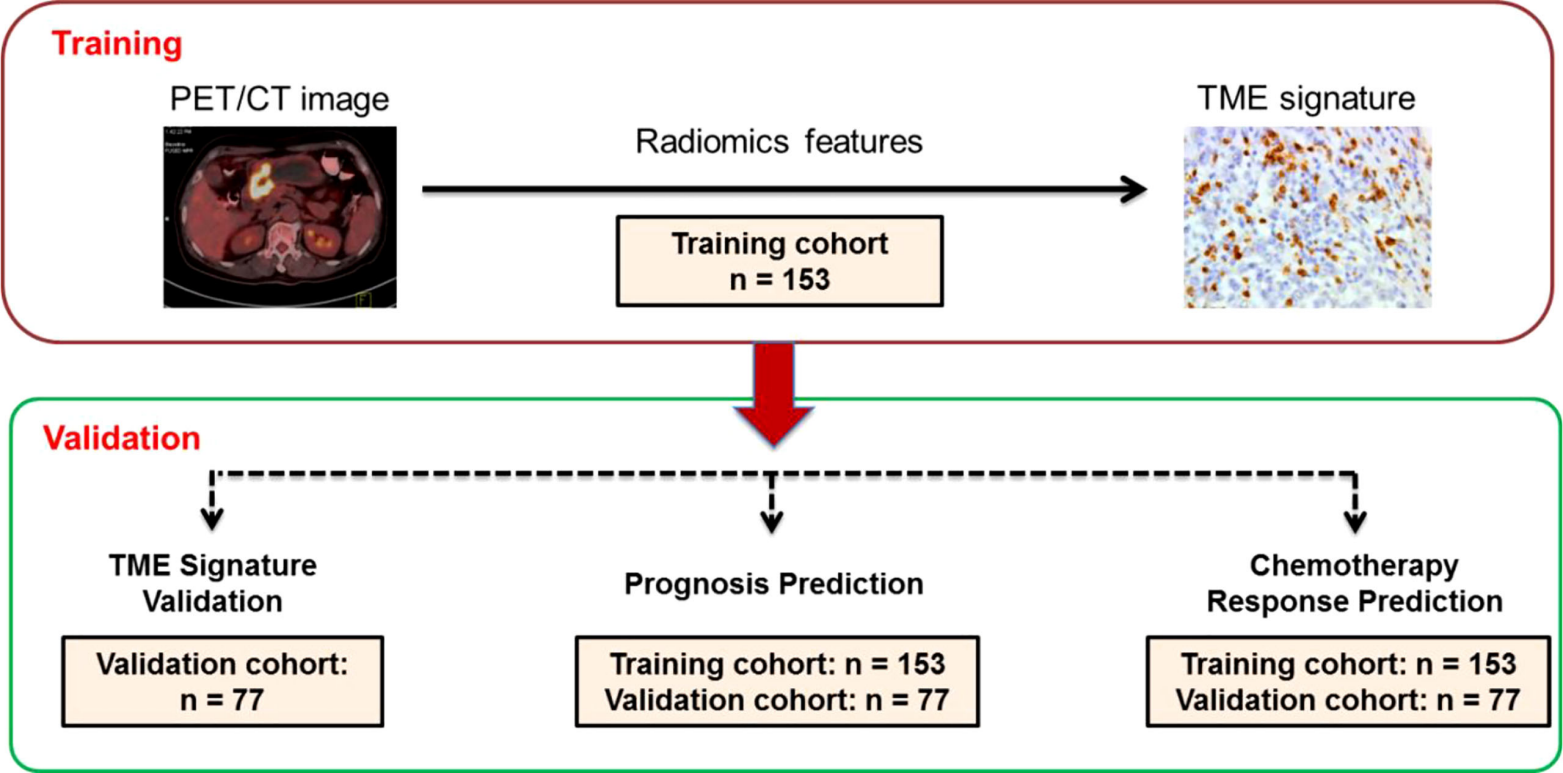
Study design for the discovery and validation of a PET/CT radiomic signature for the tumor immune microenvironment signature in gastric cancer. PET/CT image: positron emission tomography/computer tomography image. IH,: immunohistochemistry. Training cohort (n=153); Validation cohort (n=77).

Inclusion criteria were pathology-confirmed gastric cancer, 18 to 80 years old, PET/CT performed before surgery, complete follow-up data and clinicopathologic characteristics, no treatment cancer, and patient informed consent. Excluded criteria was received any anticancer therapy previous. Under approval from the institutional ethics committee, we retrospectively collected patient data and PET/CT images in the training cohort of 153 patients with GC as the inclusion criteria at Henan Provincial People’s Hospital (Zhengzhou, China) between January 2012 and December 2020. As the same inclusion criteria, we included 77 patients in the independent validation cohort at the First Affiliated Hospital of Zhengzhou University (Zhengzhou, China) between July 2010 to December 2020 (**Figure 1**). Tumor staging was reclassified with the American Joint Committee on Cancer (AJCC) TNM Staging Manual, 8^th^ Edition. All of the included patients accepted standard gastrectomy and D2 lymph node dissection accordance with the No.5 version guideline of the Japanese Gastric Cancer Association (JGCA). Disease-free survival (DFS) was defined as the time from the time of surgery until either the date of disease progression, which refers to tumor relapse, distal metastasis, or death, or until the date that the patient was last known to be free of progression. Overall survival (OS) was defined as the time to death from any cause. Clinicopathologic information for each patient with GC, including age, sex, TNM stage, tumor size, location, differentiation, lauren type, CEA, CA199, postoperative chemotherapy and follow-up data (follow-up duration and survival), and time of baseline PET/CT imaging and surgery, were collected from the clinical medical records (**Table 1**). The two institutional ethics committees approved the retrospective study respectively.

**Table 1:** Clinical characteristics of patients according to the radiomics signature in the training and external validation cohorts.

Variables	Training cohort (n =152)			Validation cohort (n = 77)		
		low RTIMS (%)	high RTIMS (%)	*P*	lowRTIMS (%)	high RTIMS (%)	*P*
**Gender**			0.772			0.93
Female	18 (38.3%)	29 (61.7%)		9 (45.0%)	11 (55.0%)	
Male	38 (35.8%)	68 (64.2%)		25 (43.9%)	32 (56.1%)	
**Age (years), median (IQR)**	61 (56-68)	57 (48-66)		57.5 (49.5-70.25)	58 (54-69)	
**Age (years)**			0.02			0.962
<60	22 (27.8%)	57 (72.2%)		18 (43.9%)	23 (56.1%)	
≧60	34 (45.9%)	40 (54.1%)		16 (44.4%)	20 (55.6%)	
**Tumor size (cm)**			0.518			0.072
<4	19 (33.3%)	38 (66.7%)		9 (31.0%)	20 (69.0%)	
≧4	37 (38.5%)	59 (61.5%)		25 (52.1%)	23 (47.9%)	
**Tumor location**			0.023			0.442
Cardia	26 (48.1%)	28 (51.9%)		14 (48.3%)	15 (51.7%)	
Body	6 (22.2%)	21 (77.8%)		2 (25.0%)	6 (75.0%)	
Antrum	15 (27.3%)	40 (72.7%)		10 (38.5%)	16 (61.5%)	
Whole	9 (52.9%)	8 (47.1%)		8 (57.1%)	6 (42.9%)	
**Differentiation status**			0.459			0.297
Well	11 (47.8%)	12 (52.2%)		5 (33.3%)	10 (66.7%)	
Moderate	10 (37.0%)	17 (63.0%)		7 (63.6%)	4 (36.4%)	
Poor and undifferentiated	35 (34.0%)	68 (66.0%)		22 (43.1%)	29 (56.9%)	
**Lauren type**			0.24			0.842
Intestinal type	28 (41.8%)	39 (58.2%)		15 (45.5%)	18 (54.5%)	
Diffuse or mixed type	28 (32.6%)	58 (67.4%)		19 (43.2%)	25 (56.8%)	
**CEA**			0.615			0.971
Normal	49 (37.4%)	82 (62.6%)		27 (40.3%)	34 (55.7%)	
Elevated	7 (31.8%)	15 (68.2%)		7 (43.8%)	9 (56.3%)	
**CA199**			0.232			0.638
Normal	43 (34.4%)	82 (65.6%)		22 (42.3%)	30 (57.7%)	
Elevated	13 (46.4%)	15 (53.6%)		12 (48.0%)	13 (52.0%)	
**Depth of invasion**			0.007			0.103
T1	1 (6.7%)	14 (93.3%)		0 (0.0%)	4 (100.0%)	
T2	6 (28.6%)	15 (71.4%)		3 (27.3%)	8 (72.7%)	
T3	2 (14.3%)	12 (85.7%)		2 (40.0%)	3 (60.0%)	
T4a	35 (43.2%)	46 (26.8%)		23 (46.9%)	26 (53.1%)	
T4b	12 (54.5%)	10 (45.5%)		6 (75.0%)	2 (25.0%)	
**Lymph node metastasis**			0.285			0.263
N0	12 (26.1%)	34 (73.9%)		8 (36.4%)	14 (63.6%)	
N1	10 (45.5%)	12 (54.5%)		7 (77.8%)	2 (22.2%)	
N2	11 (39.3%)	17 (60.7%)		5 (35.7%)	9 (64.3%)	
N3a	14 (35.0%)	26 (65.0%)		10 (41.7%)	14 (58.3%)	
N3b	9 (52.9%)	8 (47.1%)		4 (50.0%)	4 (50.0%)	
**Stage**			0.017			0.075
II	15 (25.0%)	45 (75.0%)		7 (29.2%)	17 (70.8%)	
III	41 (44.1%)	52 (55.9%)		27 (50.9%)	26 (49.1%)	
**Chemotherapy**			0.022			0.919
No	16 (25.8%)	46 (74.2%)		17 (44.7%)	21 (55.3%)	
Yes	41 (44.0%)	51 (56.0%)		17 (43.6%)	22 (56.4%)	

RTIMS, radiomics tumor immune microenvironment score.

### Immunohistochemistry (IHC) staining and classification of tumor microenvironment (TME) signature

An support vector machine (SVM) based tumor microenvironment signature integrating seven features, including CD3 _invasive margin (IM)_, CD8 _IM_, CD45RO _center of tumor (CT)_, CD66b _IM_, CD34, periostin, and cyclooxygenase-2, was previously developed and validated (7). Formalin-fixed paraffin-embedded (FFPE) samples were processed for IHC staining as previously described (7, 28–30). Following incubation with an antibody against human CD3 (pan T lymphocytes; NeoMarker, clone SP7), CD34 (Abcam, ab81289), CD8 (cytotoxic T lymphocytes; NeoMarker, clone SP16), CD45RO (memory T lymphocytes; Invitrogen, clone UCHL1) and CD66b (neutrophils; BD Pharmingen), periostin (Abcam,ab92460), and cyclooxygenase-2 (Abcam, Cambridge, MA), the sections were stained in an EnVision System (Dako) (**Table S1**). Two pathologists who were blinded to clinical outcomes independently scored all samples. If there was a difference opinion between the two primary pathologists, the third pathologist was consulted to give the final decision. As the previously described (7) and the result of IHC, every patient was classified into a high-SVM group and a low-SVM group. Detailed information is provided in the Supplementary Materials.

### PET/CT imaging

All patients underwent contrast-enhanced ^18^F fluorodeoxyglucose (FDG) PET/CT scans before surgery. Details about image acquisition and processing procedures are presented in the **Supplementary Materials**. Tumor segmentation was then performed based on agreement reached by two expert radiology physicians, and checked by authors Yang You and Huihui Guo on the PET/CT images with *ITK-SNAP* software (www.itksnap.org) (31, 32).

### Image feature extraction

We calculated a total of 80 quantitative features from each volume of interest (VOI) of each patient’s PET/CT image to characterize intratumor heterogeneity and complexity. The feature pool included 14 first-order intensity features, 9 shape features, and 57 second- and higher-order textural features. In this work, we investigated four types of texture features on the basis of gray-level co-occurrence matrices (GLCM), gray-level run length matrix (GLRLM), gray-level size zone matrix (GLSZM), and neighborhood gray-tone difference matrix wavelet decompositions (NGTDM). 26, 13, 13 and 5 features were extracted from GLCM, GLRLM, GLSZM and NGTDM, respectively. The detailed mathematical definitions of all imaging features listed in **Supplementary Materials**. All radiomic features were extracted in Matlab R2012a (The MathWorks Inc.) using an available radiomic analysis package (https://github.com/mvallieres/radiomics/).

The SUV image was discretized by 0.1 SUV unit bin width according to the following equation (33): *SUV_Dis_* (*x*) = | *SUV*(*x*)/0.1| – min(|*SUV*(*x*)/0.1|) + 1, where *SUV*(*x*) is the SUV of voxel *x* and *SUV_Dis_* (*x*) is the discretized value of voxel *x*. The discretization step is necessary to generate matrices whose size (defined by the maximum *SUV_Dis_* (*x*)) highly impacts computation, and is used to reduce image noise and generate a constant intensity resolution so that textural features from different patients are comparable.

### Construction of a radiomics tumor immune microenvironment score

We developed a logistic regression model to predict the IHC-based tumor immune microenvironment score (radiomics tumor immune microenvironment score, RTIMS) *via* a linear combination of selected image features weighted by their respective coefficients. We used the least absolute shrinkage and selection operator (LASSO) method to select the most useful predictive features from the training cohort. The diagnostic ability of the model was assessed with the area under the characteristics operating curves (AUC). The optimal cutoff value for RTIMS was determined using Youden’s index in the training cohort, which maximizes the sum of sensitivity and specificity. This cutoff value was fixed and then applied to the validation cohort. The “glmnet” package was used to perform the LASSO regression model analysis (34, 35). Complete details are provided in **Supplementary Materials**.

### Association with prognosis and chemotherapy benefits

The potential association of the RTIMS with DFS and OS was first assessed in the training cohort and then validated in the validation cohorts by using Kaplan-Meier survival analysis. Stratified analyses were performed to explore the potential association of RTIMS with DFS and OS using subgroups within TNM stage. The association between RTIMS and adjuvant chemotherapy response was assessed in patients with stage II and III GC.

### Statistical analysis

We compared two groups using the *t*-test for continuous variables and the chi-square test or Fisher exact test for categorical variables, as appropriate. Survival curves were generated using the Kaplan-Meier method and compared using the log-rank test. Univariate and multivariate analyses were performed using the Cox proportional hazards model. Variables that achieved statistical significance at *P* < 0.05 were entered into the multivariate Cox regression analyses. Interactions between the RTIMS and chemotherapy were detected using the Cox model as well. All the statistical tests were done with the SPSS software (version 21.0) and R software (version 3.5.1). A two-sided *P* value < 0.05 was considered significant.

## Results

### Clinical characteristics

**Table 1** list the detailed clinicopathological characteristics of the patients in the training (n=153) and validation (n=77) cohorts. All of the 230 patients included in the study, 163 (70.87%) were men, and the median (interquartile range, IQR) age was 58.0 (51.0-68.0) years. The number of patients with stage II or stage III GC who received adjuvant chemotherapy was 92 (60.1%) in the training cohort, 39 (50.6%) in the validation cohort.

### Development and validation of Radiomics Tumor Immune Microenvironment Score (RTIMS)

**Figure 2:**
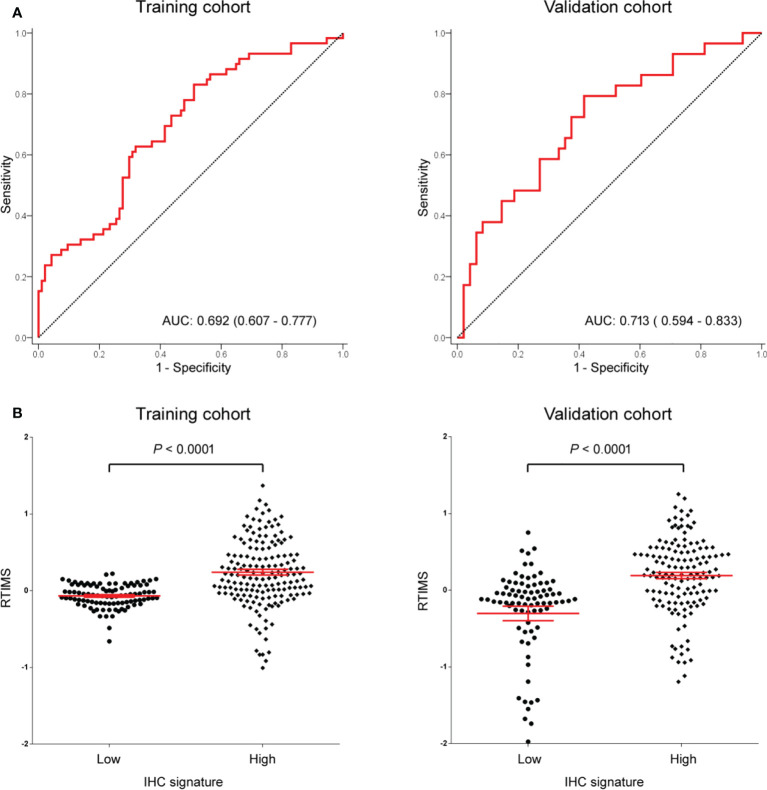
**(A)** AUC of the receiver operator characteristic of RTIMS predicting the tumor immune microenvironment signature in the training cohort and validation cohort. **(B)** RTIMS by high and low IHC signature. RTIMS, radiomics tumor immune microenvironment score. AUC, area under the curves.

In the training cohort, a LASSO logistic regression model was built. The final radiomics signature (RTIMS, Radiomics Tumor Immune Microenvironment Score) included 8 features (**Figure S1**). The RTIMS calculation formula is RTIMS = -0.006 * SUV_SD - 0.134 * Hist_Energy + 0.152 * InVar_GLCM + 0.132 * LRHGE_GLRLM + 0.019 * SZLGE_GLSZM + 0.005 * ZSV_GLSZM + 0.195 * Complexity_NGTDM + 0.003 *Contrast_NGTDM. The ability of the radiomics signature to classify high versus low-SVM was shown to have an AUC of 0.692 (95% CI 0.6077-0.777) in the training cohort (**Figure 2A**). The radiomics signature showed similar accuracy in the validation cohort with AUC: 0.713 (95% CI 0.594-0.833) (**Figure 2A**). We further confirmed that the RTIMS score was significantly higher in the high TME group than the RTIMS in the low TME group in the training cohort and validation cohort, respectively (**Figure 2B**). The optimum cutoff of RTIMS determined by the ROC curve analysis in the training cohort was -0.069 (**Figure 2A**). Accordingly, patients were classified into a low-RTIMS group (RTIMS < -0.069) and a high-RTIMS group (RTIMS ≥-0.069). **Table 1** lists the relationships between the RTIMS and clinicopathological characteristics.

### Prognostic value of RTIMS

**Figure 3:**
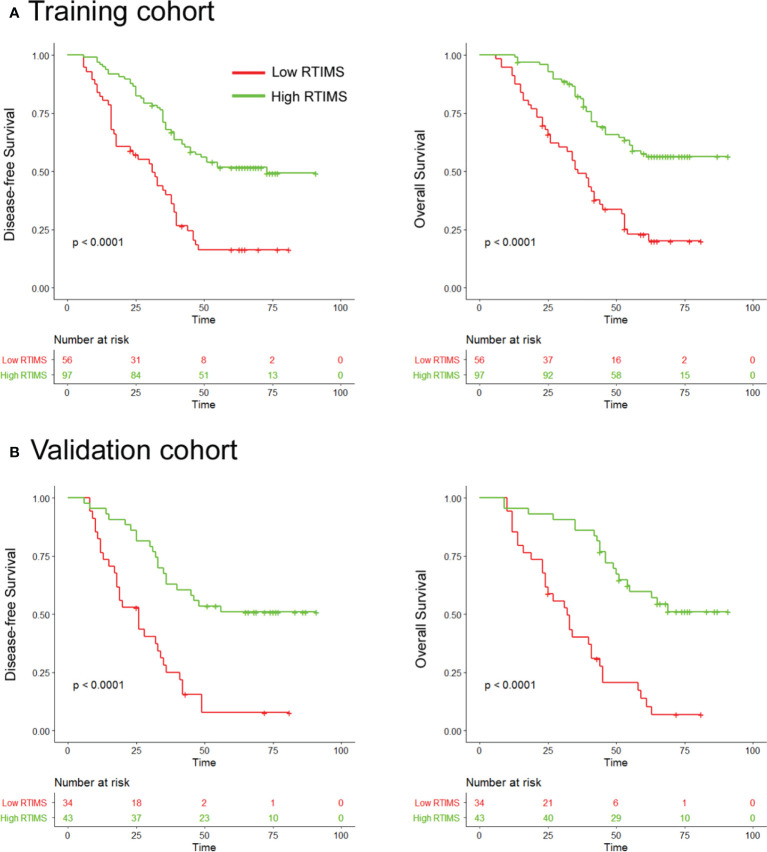
Kaplan-Meier analyses of disease-free survival (DFS) and overall survival (OS) according to dichotomized RTIMS signature in patients with gastric cancer. **(A)** Training cohort (n=153), **(B)** Validation cohort (n=77). Left panel: DFS; right panel: OS.

We first assessed the prognostic value of the RTIMS in the training cohort. For the low- RTIMS group, the 5-year DFS and OS were 16.39% and 22.73%; for the high- RTIMS group, the 5-year DFS and OS were 51.67% and 57.58% (hazard ratios (HRs) 0.361 (95%CI 0.239-0.547) and 0.291 (0.164-0.517), all *P*< 0.0001; **Figure 3A**). We then performed the same analyses in the validation cohort and found similar results. The 5-year DFS and OS were 7.8% and 13.74% for the low-RTIMS group compared with 50.94% and 59.53% for the high-RTIMS group (HRs 0.339(0.219-0.525) and 0.232 (0.129-0.419), all *P*< 0.0001; **Figure 3B**).

**Figure 4:**
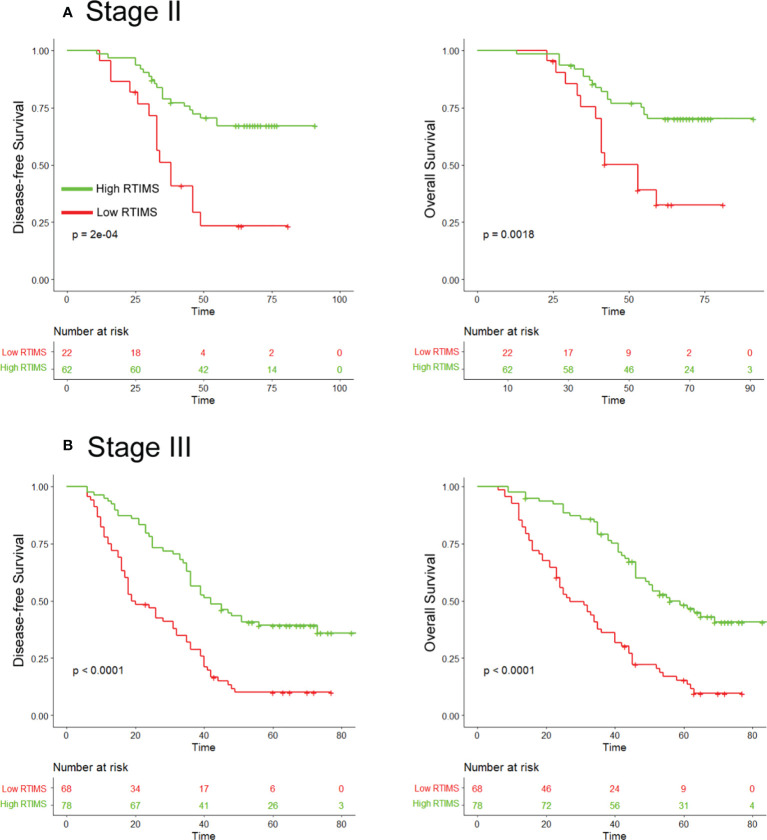
Kaplan-Meier survival analysis of disease-free survival and overall survival according to the RTIMS signature in subgroups of stage II and III GC patients. Disease-free survival (left pane) and overall survival (right pane): **(A)** Stage II (n = 84), **(B)** Stage III (n = 146).

Univariate Cox regression analysis and multivariate Cox regression analysis were performed adjusting for clinicopathological variables. The RTIMS remained a powerful and independent prognostic factor for predicting DFS and OS in the training and validation cohorts (**Table 2** and **Table S2**). Finally, we performed additional analyses within subgroups of GC patients who are stratified by stage. High-RTIMS patients had a longer DFS and OS than patients with low-RTIMS within each stage II or III (**Figure 4**).

**Table 2:** Multivariate Cox Regression analyses for disease-free survival and overall survival in the training cohort of patients with gastric cancer.

Variables	Disease-free survival		Overall survival	
	HR (95%CI)	*p*	HR (95%CI)	*p*
**Training cohort**				
RTIMS(High vs. low)	0.404(0.266-0.615)	<0.0001	0.336(0.215-0.526)	<0.0001
Stage(III vs. II)	2.145(1.346-3.420)	0.001	2.036(1.219-3.403)	0.007
Differentiation	/	/	1.413(1.009-1.979)	0.044
**Validation cohort**				
RTIMS (High vs. low)	0.275(0.150-0.506)	<0.0001	0.198(0.107-0.367)	<0.0001
Stage(III vs. II)	2.066(1.020-4.184)	0.044	/	/
CEA	2.103(1.072-4.124)	0.031	2.446(1.264-4.731)	0.008

RTIMS, radiomics tumor immune microenvironment score.

### Predictive value of RTIMS for chemotherapy benefits

**Figure 5:**
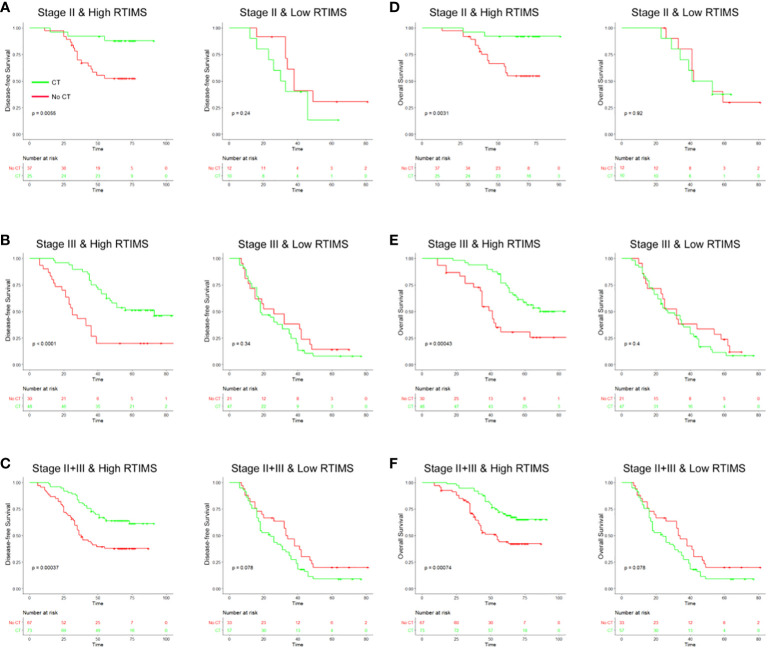
Relationship between the RTIMS signature and survival benefit from adjuvant chemotherapy in patients with stage II and III gastric cancer. In stage II, stage III and stage II+III patients, adjuvant chemotherapy improved DFS **(A–C)** and OS **(D–F)** for patients with high RTIMS, whereas no effect on survival was observed in patients with low RTIMS. RTIMS, radiomics tumor immune microenvironment score.

Then, we evaluated the relationship between RTIMS status and survival among stage II and III patients who either received or did not receive postoperative chemotherapy. The characteristics of patients who received adjuvant chemotherapy were similar to those of patients who did not receive adjuvant chemotherapy (**Table S3**). The results showed that adjuvant chemotherapy was associated with an improved prognosis in the high-RTIMS group for both stage II and III disease, e.g., for DFS, stage II: HR 0.208(95% CI 0.061-0.711), p=0.005; stage III: HR 0.321(0.180-0.570), p<0.001 (**Figure 5**). However, for patients in the low-RTIMS group, chemotherapy did not affect survival in either stage II or III disease: for DFS, stage II: HR 1.799(0.646-5.009), p=0.240; stage III: HR 1.302(0.744-2.277), p=0.340. Then, a formal interaction test was performed between the RTIMS signature and chemotherapy, which confirmed a significant interaction regarding the impact on DFS and OS in stage II disease (p for interaction: DFS, p=0.008; OS, p=0.034) as well as in stage III disease (p for interaction: DFS, p=0.001; OS, p=0.004). This analysis suggests a predictive effect of RTIMS for the benefit of adjuvant chemotherapy.

## Discussion

Radiomics approaches, when combined with tumor biopsies and genomics, could improve treatment selection. Radiomic features from tumor can provide information on both the tumor and its microenvironment (18, 36). In our study, we developed and validated a radiomic signature (RTIMS) of the tumor immune microenvironment, and the RTIMS signature was able to predict survival. Moreover, the RTIMS signature might help to identify stage II and III patients who could benefit from adjuvant chemotherapy.

The importance of radiomics is shown by the increasing number of oncological clinical trials being done that use radiomics. To date, more than 30 clinical studies are registered in ClinicalTrials.gov, including one prospective study of pembrolizumab (NCT02644369). Notably, Braman et al. (18) that evaluated radiomic features in the context of neoadjuvant chemotherapy for breast cancer found radiomics features could strongly predict pCR independent of choice of classifier, suggesting their robustness as response predictors. Moreover, pathological complete response was associated with infiltration of lymphocytes into tumors (18) Jiang et al. measure immune score based on the radiomics features from CT image (17). And, the CT image signature could predict survival and treatment response, which is concordant with our results.

Notably, several points strengthening the biological and clinical relevance of this radiomic signature have been identified. Grossmann et al. discovered a connection between the radiomic phenotype of a tumor, the signaling pathways inside cells that drive how cancer develops, and clinical treatment outcomes (14). They also did IHC staining of CD3 in 22 tumors that were predicted to show relatively high or low immune response based on one radiomic feature, and they found agreement between radiomic features and pathology. Thus, they deemed that radiomic approaches permit noninvasive assessment of both molecular and clinical characteristics of tumors, however, the results still should be interpreted with caution. Ferté et al. also developed a radiomic signature for the gene expression signature of CD8 cells, which could predict clinical outcomes in patients treated with anti-PD-1 or anti-PD-L1 immunotherapy (15).

At present, the standard treatment for advanced gastric cancer includes adjuvant chemotherapy after surgery to prevent disease recurrence and improve survival, however, many studies have reported that a subgroup of patients could not benefit from adjuvant chemotherapy (25, 26, 28). Moreover, the criteria for the selection of candidates who are more likely to benefit from adjuvant chemotherapy remain controversial. Thus, the accurate identification of subgroups of patients will improve the prognostic system and lead to more personalized therapy. Recently, several studies reported that radiomics signatures based on CT/MRI/PET images were associated with chemotherapy response in several types of cancers (37–39). Besides, Ferté et al. and Jiang et al. previous study showed that imaging biomarkers could be used to estimate tumor-infiltrating lymphocytes (15, 16), which were associated with chemotherapy response (28, 40). In this study, we found that adjuvant chemotherapy provided a better survival benefit to patients with stage II and III GC patients classified as high-RTIMS, whereas low-RTIMS patients did not obtain benefits from adjuvant chemotherapy; further use of the radiomics signature might allow for better identification of patients who are most likely to benefit from adjuvant therapy. Thus, we suggest that patients with low-RTIMS should be treated with new combinations of more tolerable medication as an adjunct to potentiate the efficacy of systemic approaches. Therefore, our PET/CT image-based RTIMS signature for patients with stage II and III gastric cancer is both a prognostic and predictive method, in that patients with a high- RTIMS have a clear benefit from adjuvant chemotherapy.

Interpretation of complex images by radio-pathology/genomic approaches is currently changing several fields in medical imaging, but clinical application of this method is still in its infancy. Validating this approach in a separate cohort, we confirmed the prognostic power of this approach. Thus, A novel biomarker that can be incorporated into existing clinical workflows because it only relies on PET/CT images, which are non-invasive and widely available. Compared to the gene-based signatures, a major advantage of our radiomics method is the ubiquitous availability of PET/CT images, which are available for every cancer patient, and analyzing them is not very costly. Besides, abdominal PET/CT scans are effective tools to diagnose and guide treatment for patients with gastric cancer. Whereas, those gene-based signatures have not been widely led into clinical application as initially expected owing to the variability of measurements in microarray and sequencing assays, inconsistencies in assay platforms, and the requirement for analytical expertise (41–43). In addition, our radiomic approach of the TME signature is reproducible: If presented with the same image twice, the algorithm will export the same result. These points make this new approach well suited for a clinical application.

According to the molecular classification of The Cancer Genome Atlas (TCGA) project, gastric cancer was divided into four subtypes: Epstein-Barr virus (EBV)-positive, microsatellite instability (MSI), genomic stability, and chromosomal instability (44). High lymphocytes infiltration were frequently observed in certain molecular subtypes of GC, such as EBV-positive and MSI subtypes (45, 46). According to a phase II study, mismatch repair deficiency or MSI-high renders different solid tumors highly sensitive to immune checkpoint blockade with the PD-1 inhibitor pembrolizumab, and these tumors contain prominent lymphocytes infiltration (47, 48). Recently, several studies showed that the tumor imaging biomarkers could provide a promising way to predict the immune phenotype of tumors and to infer clinical outcomes for patients with cancer who had been treated with PD-1 and PD-L1 inhibitor (15, 16). Therefore, future studies should investigate the association between the radiomic signature of tumor immune microenvironment and molecular classification, and explore whether the radiomic signature could predict the responses of patients with gastric cancer to immunotherapy.

There are some limitations to this study. First, the study was conducted retrospectively, which was susceptible to the inherent biases of such a study format. Secondly, the decision to accept postoperative chemotherapy or not was made by the patients and clinicians together, that was not within a randomized assignment. Thirdly, the model was developed and validated using data from East Asian patients, and its generalizability in western populations remains to be determined. Therefore, a prospective, international, multicenter clinical trial will be needed to further validate our findings.

In conclusion, we developed a PET/CT image based radiomic signature that allows the noninvasive evaluation of tumor immune microenvironment. Moreover, the RTIMS might be a useful predictive tool to identify stage II and III patients benefit from adjuvant chemotherapy. Thus, the RTIMS potentially may offer clinical value in directing individualized therapeutic regimen selection for patients with stage II and III gastric cancer.

## Data availability statement

The original contributions presented in the study are included in the article/**Supplementary Material**. Further inquiries can be directed to the corresponding author.

## Ethics statement

The studies involving human participants were reviewed and approved by Ethics Committee of Henan Provincial Hospital. The patients/participants provided their written informed consent to participate in this study. Written informed consent was obtained from the individual(s) for the publication of any potentially identifiable images or data included in this article.

## Author contributions

Conception and design: JL and JB. Collection and assembly of data: JL, HW, HZ, and SL. Data analysis and interpretation: CZ, YY, HG, and PZ. Manuscript writing: all authors. Final approval of manuscript: all authors.

## Funding

This study was supported by the Project of Tackle Key Problems in Science and Technology of Henan Province (No.212102310679), and the Project Construction of National Key Disciplines (General Surgery Department, 2021).

## Conflict of interest

The authors declare that the research was conducted in the absence of any commercial or financial relationships that could be construed as a potential conflict of interest.

## Publisher’s note

All claims expressed in this article are solely those of the authors and do not necessarily represent those of their affiliated organizations, or those of the publisher, the editors and the reviewers. Any product that may be evaluated in this article, or claim that may be made by its manufacturer, is not guaranteed or endorsed by the publisher.
