# Supplementary material for: Non-invasive measurement of tumor immune microenvironment and prediction of survival and chemotherapeutic benefits from 18F fluorodeoxyglucose PET/CT images in gastric cancer
Source: Front Immunol. 2022 Oct 13;13:1019386. doi: 10.3389/fimmu.2022.1019386 (PMC9606753; doi:10.3389/fimmu.2022.1019386)
Supplement: Supplementary file 1 [file DataSheet_1.docx]

**Supplementary Materials**

- **Supplementary Methods**
- **Supplementary Reference**
- **Supplementary Tables**
- **Supplementary Figures**
- **Definition of image features**

**Supplementary Methods**

**1. Immunohistochemistry (IHC) staining and classification of TME signature**

Formalin-fixed paraffin-embedded (FFPE) samples were processed for IHC staining as previously described [[1-3](#_ENREF_1)]. The antibody dilutions and antigen retrieval are shown in Table S1**.** Every staining run contained a slide treated with phosphate buffer saline (PBS) buffer in place of the primary antibody as a negative control. Every staining run contained a slide of positive control. Prior to staining, sections were blocked with endogenous peroxidase (prepared in 1% H_2_O_2_/methanol solution) for 10 minutes and then microwaved for 30 minutes in 10 mM citrate buffer, pH 6.0. The sections were blocked using 10% normal rabbit serum for 30 minutes. Furthermore, all slides were stained with the same concentrations of primary antibody for each antibody and incubated with monoclonal primary antibody overnight at 4 ℃, followed by incubation with an amplification system with a labeled polymer/HRP (EnVision™, DakoCytomation, Denmark) at 37℃ for 30 minutes. The sections were developed with 0.05% 3, 3´-diaminobenzidine tetrahydrochloride (DAB) and counterstained with modified Harris hematoxylin. And all slides were stained with DAB dyeing for the same time for each antibody (Table S1). The IHC results were evaluated by 2 independent observers who were blinded to the clinical outcome. A third pathologist was consulted when a difference of opinion arose between the 2 primary pathologists. At low power (100), the tissue sections were screened using an inverted research microscope (model DM IRB; Leica, Germeny), and the 5 most representative fields were selected. Thereafter, to evaluate the density of stained immune cells, the 2 respective areas of invasive margin and center of tumor were measured at 200 magnification. The nucleated stained cells in each area were quantified and expressed as the number of cells per field.

SVM is a powerful method for building a classifier. It aims to create a decision boundary between two classes that enables the prediction of labels from one or more feature vectors.[[4](#_ENREF_4)] This decision boundary, known as the hyperplane, is orientated in such a way that it is as far as possible from the closest data points from each of the classes. These closest points are called support vectors. In this study, as previously described [[5](#_ENREF_5)], every patient was classified into a high-SVM group and a low-SVM group based on seven features, including CD3 IM, CD8 IM, CD45RO CT, CD66b IM, CD34, POSTN, and COX2. The programs were coded using R software; scripts are available on request.

**2. PET/CT imaging**

Patients with GC who underwent PET/CT were imaged after a 6-8 h fast and checked for a blood glucose level in the range of 3.6-10 mM. The blood glucose level was monitored by finger stick immediately before the injection of ^18^F FDG. ^18^F FDG with a radiochemical purity greater than 95% was manufactured automatically using the tracer synthesis system of a Tracerlab FXF-N (GE Healthcare). Patients were injected with 161–361 MBq (4.35-9.76 mCi, 150 μCi/kg), and imaging was performed 60 min (59 ± 3 min, range 53-62 min) later with a PET/CT scanner (GE Discovery LS PET/CT scanner (GE Healthcare, Waukesha, Wisconsin, USA)). CT images were collected in the helical acquisition mode. In the same scanning locations and generally in 6-8 bed positions, PET data were acquired with 3-5 min of acquisition time per bed position. The complete PET/CT examination required approximately 1.5 h, including patient setup, tracer uptake, and image acquisition.

PET images were reconstructed using standard ordered-subset expectation maximization (OSEM). The reconstruction thicknesses of the CT images were 4.25 mm. The field of views (FOV) were 700 mm, and matrix sizes were 512×512. The OSEM algorithm (3 iterations and 21 subsets) was used for PET image reconstruction, resulting in voxel sizes of and 4.3×4.3×4.25 mm^3^. The CT image voxel sizes were 1.95×1.95×5 mm^3^. Images were corrected for attenuation with a CT-based attenuation correction method. The PET and CT images were individually transferred to Xeleris workstations, respectively, to display frame-on-frame fusion images.

**3. Construction of Radiomic Signature using LASSO Logistic Regression Model**

The least absolute shrinkage and selection operator method (LASSO) is a popular method for regression of high-dimensional predictors[[6-9](#_ENREF_6)]. The method uses an L1 penalty to shrink some regression coefficients to exactly zero. We plotted the AUC versus log (λ), where λ is the tuning parameter for the LASSO logistic regression model. A value of λ= 0.08209437 with log (λ) = -2.499886 was selected by maximizing AUC values in 10-fold cross validation. A vertical line was drawn at log (λ) = -2.499886, which corresponded to the optimal value λ=0.08209437 (Figure S1). The optimal tuning parameter resulted in thirteen non-zero coefficients in the final logistic regression model. The following thirteen features were selected in the LASSO logistic regression model: SUV_SD, Hist_Energy, InVar_GLCM, LRHGE_GLRLM, SZLGE_GLSZM, ZSV_GLSZM, Complexity_NGTDM, Contrast_NGTDM, with coefficients -0.005528512, -0.133634559, 0.151855772, 0.130789696, 0.019215607, 0.005201178, 0.195155689, 0.002845767.

**Supplementary Reference**

1. Jiang Y, Zhang Q, Hu Y et al. ImmunoScore Signature: A Prognostic and Predictive Tool in Gastric Cancer. Ann Surg 2018; 267: 504-513.

2. Jiang Y, Xie J, Han Z et al. Immunomarker Support Vector Machine Classifier for Prediction of Gastric Cancer Survival and Adjuvant Chemotherapeutic Benefit. Clin Cancer Res 2018; 24: 5574-5584.

3. Jiang Y, Liu W, Li T et al. Prognostic and Predictive Value of p21-activated Kinase 6 Associated Support Vector Machine Classifier in Gastric Cancer Treated by 5-fluorouracil/Oxaliplatin Chemotherapy. EBioMedicine 2017; 22: 78-88.

4. Noble WS. What is a support vector machine? Nat Biotechnol 2006; 24: 1565-1567.

5. Jiang Y, Xie J, Huang W et al. Tumor Immune Microenvironment and Chemosensitivity Signature for Predicting Response to Chemotherapy in Gastric Cancer. Cancer Immunol Res 2019; 7: 2065-2073.

6. Jiang Y, Chen C, Xie J et al. Radiomics signature of computed tomography imaging for prediction of survival and chemotherapeutic benefits in gastric cancer. EBioMedicine 2018; 36: 171-182.

7. Tibshirani R. The lasso method for variable selection in the Cox model. Stat Med 1997; 16: 385-395.

8. Tibshirani R. Regression shrinkage and selection via the lasso: a retrospective. Journal of the Royal Statistical Society Series B-Statistical Methodology 2011; 73: 273-282.

9. Zhang JX, Song W, Chen ZH et al. Prognostic and predictive value of a microRNA signature in stage II colon cancer: a microRNA expression analysis. Lancet Oncol 2013; 14: 1295-1306.

**Table S1. Antibody sources and staining conditions.**

| Markers | Antibody source | Species | Dilution | DAB dyeing time | Antigen Retrieval | Cellular  localization |
| --- | --- | --- | --- | --- | --- | --- |
| CD3(pan T cell) | NeoMarker, clone SP7 | Rabbit monoclonal | 1:300 | 1.0 min | Citrate buffer (pH 6.0) microwave 20min | Membranous |
| CD8(cytotoxic T cell) | NeoMarker, clone SP16 | Rabbit monoclonal | 1:200 | 1.5 min | Citrate buffer (pH 6.0) microwave 20min | Membranous |
| CD45RO (Memory T cell) | Invitrogen, clone UCHL1 | Mouse monoclonal | 1:400 | 1.0 min | Citrate buffer (pH 6.0) microwave 20min | Membranous |
| CD66b (Neutrophil) | BD Pharmingen | Mouse monoclonal | 1:200 | 1.0 min | Citrate buffer (pH 6.0) microwave 20min | Membranous |
| CD34(Microvessel) | Abcam, ab81289 | Rabbit monoclonal | 1:200 | 1.0 min | Citrate buffer (pH 6.0) microwave 20min | Membranous |
| POSTN | Abcam,ab92460 | Rabbit monoclonal | 1:200 | 30 secs | Citrate buffer (pH 6.0) microwave 20min | Cytoplasmic |
| COX2 | Abcam, Cambridge, MA | Mouse monoclonal | 1:200 | 30 secs | Citrate buffer (pH 6.0) microwave 20min | Cytoplasmic |

min: minute; sec: second. DAB: diaminobenzidine.

| **Table S2**. Univariate association of Rad-score, clinicopathological characteristics with disease-free and overall survival in the training and validation cohorts. | | | | | |
| --- | --- | --- | --- | --- | --- |
| **Variables** | **Training cohort** | |  | **Validation cohort** | |
|  | HR (95%CI) | *p* |  | HR (95%CI) | *p* |
| **Disease-free survival** |  |  |  |  |  |
| **RTIMS** | **0.361 (0.239-0.547)** | **<0.0001** |  | **0.291 (0.164-0.517)** | **<0.0001** |
| Age(years) (≥60 vs. <60) | 1.576 (1.044-2.380) | 0.031 |  | 1.249 (0.721-2.165) | 0.428 |
| Gender (male vs. female) | 1.052 (0.668-1.658) | 0.827 |  | 1.396 (0.716-2.723) | 0.327 |
| Tumor size (>4 cm vs. ≤4 cm) | 1.499 (0.971-2.313) | 0.068 |  | 1.726 (0.951-3.130) | 0.072 |
| Tumor location | 1.044 (0.853-1.279) | 0.676 |  | 1.171 (0.898-1.525) | 0.244 |
| Differentiation | 1.307 (0.972-1.759) | 0.076 |  | 1.014 (0.724-1.421) | 0.934 |
| Lauren type | 0.898 (0.596-1.354) | 0.609 |  | 1.143 (0.654-1.998) | 0.639 |
| CEA(ng*/*ml) | 1.726 (1.018-2.928) | 0.043 |  | 1.873 (0.997-3.522) | 0.051 |
| CA199(U*/*ml) | 1.975 (1.218-3.202) | 0.006 |  | 2.082 (1.186-3.653) | 0.011 |
| Depth of invasion | 1.442 (1.229-1.693) | <0.0001 |  | 1.409 (1.109-1.789) | 0.005 |
| Lymph node metastasis | 1.465 (1.253-1.713) | <0.0001 |  | 1.230 (1.007-1.503) | 0.043 |
| Chemotherapy | 0.911 (0.601-1.380) | 0.659 |  | 0.888 (0.572-1.378) | 0.595 |
|  |  |  |  |  |  |
| **Overall survival** |  |  |  |  |  |
| **RTIMS** | **0.339(0.219-0.525)** | **<0.0001** |  | **0.232 (0.129-0.419)** | **<0.0001** |
| Age(years) (≥60 vs. <60) | 1.573 (1.017-2.433) | 0.042 |  | 1.258(0.722-2.192) | 0.418 |
| Gender (male vs. female) | 0.943 (0.589-1.511) | 0.809 |  | 1.249 (0.639-2.440) | 0.516 |
| Tumor size (>4 cm vs. ≤4 cm) | 1.620 (1.017-2.582) | 0.042 |  | 1.519 (0.837-2.758) | 0.169 |
| Tumor location | 1.045 (0.845-1.292) | 0.685 |  | 1.205 (0.929-1.563) | 0.16 |
| Differentiation | 1.416 (1.026-1.955) | 0.034 |  | 0.972 (0.691-1.368) | 0.871 |
| Lauren type | 0.906(0.586-1.400) | 0.657 |  | 1.127 (0.642-1.978) | 0.676 |
| CEA(ng*/*ml) | 1.517 (0.853-2.701) | 0.156 |  | 1.754(0.931-3.303) | 0.082 |
| CA199(U*/*ml) | 2.048(1.233-3.404) | 0.006 |  | 1.756 (0.995-3.097) | 0.052 |
| Depth of invasion | 1.402 (1.185-1.658) | <0.0001 |  | 1.376 (1.087-1.743) | 0.008 |
| Lymph node metastasis | 1.498 (1.270-1.767) | <0.0001 |  | 1.179 (0.965-1.441) | 0.107 |
| Chemotherapy | 0.681 (0.391-1.185) | 0.174 |  | 0.698 (0.399-1.220) | 0.207 |

RTIMS, radiomics tumor immune microenvironment score.

| **Table S3. Clinical characteristics of patients according to the chemotherapy in stage II and III patients.** | | | | | | | |
| --- | --- | --- | --- | --- | --- | --- | --- |
| **Variables** | **Number** | **No chemotherapy (N=100)** | |  | **Chemotherapy (N=130)** | | ***P*-value** |
|  |  | **No.** | **%** |  | **No.** | **%** |  |
| **Gender** |  |  |  |  |  |  | 0.401 |
| Female | 67 | 32 | 32.00% |  | 35 | 26.92% |  |
| Male | 163 | 68 | 68.00% |  | 95 | 73.08% |  |
| **Age(years), median(IQR)** |  | 60(53-70) | |  | 58(50-65) | |  |
| **Age(years)** |  |  |  |  |  |  | 0.266 |
| ＜60 | 120 | 48 | 48.00% |  | 72 | 55.38% |  |
| ≧60 | 110 | 52 | 52.00% |  | 58 | 44.62% |  |
| **Tumor size(cm)** |  |  |  |  |  |  | 0.069 |
| ＜4 | 86 | 44 | 44.00% |  | 42 | 32.31% |  |
| ≧4 | 144 | 56 | 56.00% |  | 88 | 67.69% |  |
| **Tumor location** |  |  |  |  |  |  | 0.658 |
| Cardia | 83 | 32 | 32.00% |  | 51 | 39.23% |  |
| Body | 35 | 17 | 17.00% |  | 18 | 13.85% |  |
| Antrum | 81 | 38 | 38.00% |  | 43 | 33.08% |  |
| Whole | 31 | 13 | 13.00% |  | 18 | 13.85% |  |
| **Differentiation status** |  |  |  |  |  |  | 0.269 |
| Well+Moderate | 76 | 36 | 36.00% |  | 40 | 30.77% |  |
| Poor and undifferentiated | 154 | 64 | 64.00% |  | 90 | 69.23% |  |
| **Lauren type** |  |  |  |  |  |  | 0.138 |
| Intestinal type | 100 | 49 | 49.00% |  | 51 | 39.23% |  |
| Diffuse or mixed type | 130 | 51 | 51.00% |  | 79 | 60.77% |  |
| **CEA** |  |  |  |  |  |  | 0.864 |
| Normal | 192 | 83 | 83.00% |  | 109 | 83.85% |  |
| Elevated | 38 | 17 | 17.00% |  | 21 | 16.15% |  |
| **CA199** |  |  |  |  |  |  | 0.742 |
| Normal | 177 | 78 | 78.00% |  | 99 | 76.15% |  |
| Elevated | 53 | 22 | 22.00% |  | 31 | 23.85% |  |
| **Depth of invasion** |  |  |  |  |  |  | 0.007 |
| T1 | 19 | 14 | 14.00% |  | 5 | 3.85% |  |
| T2 | 32 | 19 | 19.00% |  | 13 | 10.00% |  |
| T3 | 19 | 9 | 9.00% |  | 10 | 7.69% |  |
| T4a | 130 | 48 | 48.00% |  | 82 | 63.08% |  |
| T4b | 30 | 10 | 10.00% |  | 20 | 15.38% |  |
| **Lymph node metastasis** |  |  |  |  |  |  | 0.001 |
| N0 | 68 | 44 | 44.00% |  | 24 | 18.46% |  |
| N1 | 31 | 12 | 12.00% |  | 19 | 14.62% |  |
| N2 | 42 | 14 | 14.00% |  | 28 | 21.54% |  |
| N3a | 64 | 21 | 21.00% |  | 43 | 33.08% |  |
| N3b | 25 | 9 | 9.00% |  | 16 | 12.31% |  |
| **Stage** |  |  |  |  |  |  | 0.001 |
| II | 84 | 49 | 49.00% |  | 35 | 26.92% |  |
| III | 146 | 51 | 51.00% |  | 95 | 73.08% |  |
| **RTIMS** |  |  |  |  |  |  | 0.095 |
| Low | 90 | 33 | 33.00% |  | 57 | 43.85% |  |
| High | 140 | 67 | 67.00% |  | 73 | 56.15% |  |

RTIMS, radiomics tumor immune microenvironment score.


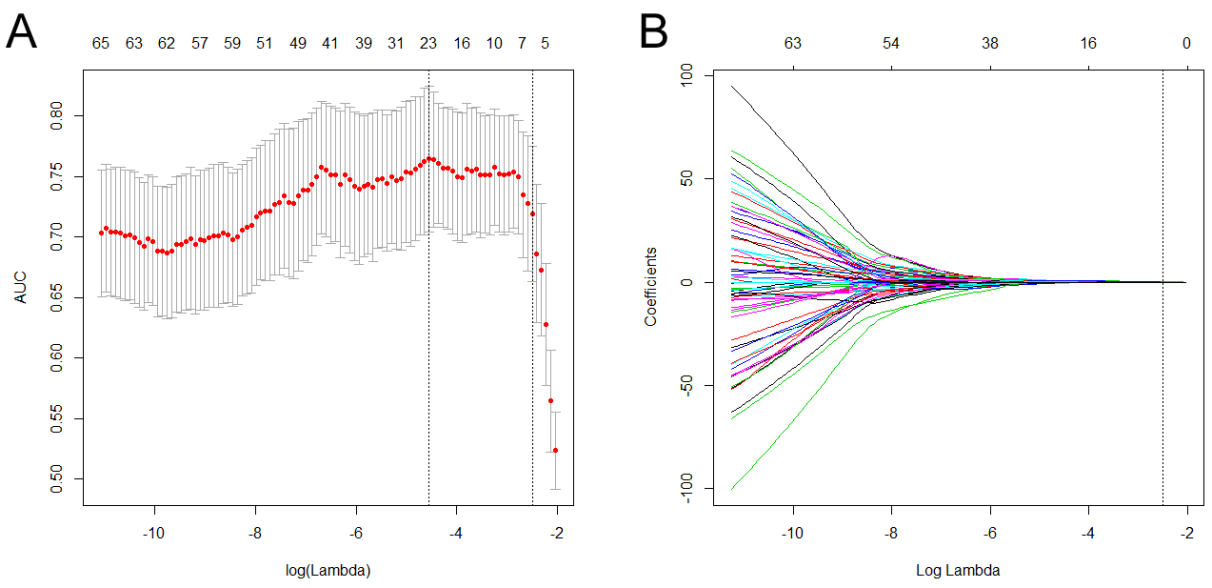


**Figure S1. Texture feature selection using the least absolute shrinkage and selection operator (LASSO) logistic regression model.** (A) Tuning parameter (λ) selection in the LASSO model used 10-fold cross-validation via minimum criteria. The partial likelihood deviance (PLD) curve was plotted versus log (λ). Dotted vertical lines were drawn at the optimal values by using the minimum criteria and 1 standard error of the minimum criteria (the 1-SE criteria). A λ value of 0.08209437, with log (λ) of -2.499886 was chosen (1-SE criteria) according to 10-fold cross-validation. (B) LASSO coefficient profiles of the 80 texture features. A coefficient profile plot was produced against the log (λ) sequence. A vertical line was drawn at the value selected using 10-fold cross-validation, where optimal λ resulted in eight nonzero coefficients.

**Definition of image features**

- ***Intensity features-15***

Let $P$ define the first-order histogram of tumor volume. $P(i)$ represents the number of voxels with SUV values of $i$, and $N_{g}$ represents the number of gray-level bins set for $P$. The $i^{th}$ entry of the normalized histogram is then defined as:

$$p(i)=\frac{P(i)}{\sum_{i=1}^{N_{g}} P(i)}$$

1. SUV_max: the maximum SUV value.
2. SUV_mean: the mean SUV value.
3. SUV_min: the minimum SUV value.
4. SUV_median: the median SUV value.
5. SUV_range: then range of SUV value.
6. SUV_MAD: Mean absolute deviation, the mean of the absolute deviations of all voxel SUVs around the mean SUV value.
7. SUV_SD: the standard deviation of all SUV values.
8. SUV_RMS: root mean square, the quadratic mean, or the square root of the mean of squares of all voxel SUVs.

$$RMS=\sqrt{\frac{\sum_{i=1}^{N_{g}} i^{2}}{N_{g}}}$$

1. Hist_mean:

$$\mu=\sum_{i=1}^{Ng} ip(i)$$

1. Hist_Var:

$$\sigma^{2}=\sum_{i=1}^{N_{g}} (i-\mu)^{2}p(i)$$

1. Hist_Skewness:

$$s=\sigma^{-3}\sum_{i=1}^{N_{g}} (i-\mu)^{3}p(i)$$

1. Hist_Kurtosis:

$$k=\sigma^{-4}\sum_{i=1}^{N_{g}} (i-\mu)^{4}p(i)-3$$

1. Hist_Energy:

$$energy\_hist=\sum_{i=1}^{N_{g}} p(i)^{2}$$

1. Hist_Entropy:

$$entropy\_hist=-\sum_{i=1}^{Ng} p(i)\log_{2}[p(i)]$$

1. TLG: total lesion glycolysis, defined as the product of MATV and SUVmean.

- ***Shape features-9***

Shape features, describing the shape and size of the volume of interest. Let $M$ as the number of voxels in the tumor.

1. MATV: metabolically active tumor volume (V)

$$MATV=M\times size\text{ }voxel\text{ }volume\text{ }$$

1. Surface: the surface area of the volume of interest (A).
2. Compactness 1:

$$compactness 1=\frac{\text{V}}{\sqrt{\pi}\text{A}^{\frac{2}{3}}}$$

1. Compactness 2:

$$compactness 2=36\pi\frac{V^{2}}{\text{A}^{3}}$$

1. Sphericity：

$$sphericity=\frac{\pi^{\frac{1}{3}}\left(6V\right)^{\frac{2}{3}}}{\text{A}}$$

1. SVratio: the surface area divided by the volume.
2. Irregularity:

$$irregularity=\frac{A}{3\cdot4\pi(\frac{V}{4\pi})^{\frac{2}{3}}}$$

1. Eccentricity: find an ellipsoid that best fits the tumor region, and the eccentricity is then given by $(1-a\times\frac{b}{c^{2}})^{\frac{1}{2}}$, where $c$ is the longest semi-principal axes of the ellipsoid, $a$ and $b$ are the second and third longest semi-principal axes of the ellipsoid.
2. Solidity: ratio of the number of voxels in the tumor region to the number of voxels in the 3D convex hull of the tumor region (smallest polyhedron containing the tumor region).

- ***Gray Level Co-occurrence Matrix-based features (GLCM)-26***

Gray level co-occurrence matrix-based features, as described by study [1]. The element $P(i,j)$ of normalized co-occurrence matrix represent the number of times that intensity $\text{i}$ and $\text{j}$ appeared in two voxels separated by distance D in direction $\theta$. The co-occurrence matrix is given by:

$$P(i,j)=\#\left\{(I(\text{x},\text{y},\text{z})=\text{i},\text{I}(\text{k},\text{l},\text{m})=\text{j})\text{ | D},\theta\right\}$$

where # represents the number of times, $I$ represents the voxel intensity, $(\text{x},\text{y},\text{z})$ and $(\text{k},\text{l},\text{m})$ are the coordinates (positions) of two different voxels, the direction vector is thus determined by $(\text{k},\text{l},\text{m})-(\text{x},\text{y},\text{z})$, $N_{g}$ is the number of discrete intensity levels in the image, and $\mu$ is the mean of $P(i,j)$. The feature is derived by considering all the 13 directions simultaneously, thus arriving at a single matrix.

Let us define:

$$\mu_{x}\text{=}\sum_{\text{i}=1}^{\text{N}_{\text{g}}} i\sum_{j=1}^{Ng} P(i,j)$$

$$\mu_{\text{y}}\text{=}\sum_{\text{j}=1}^{\text{N}_{\text{g}}} j\sum_{i=1}^{Ng} P(i,j)$$

$$\sigma_{x}\text{=}\sqrt{\sum_{\text{i}=1}^{\text{N}_{\text{g}}} (i-\mu_{x})^{2}\sum_{j=1}^{Ng} P(i,j)}$$

$$\sigma_{y}\text{=}\sqrt{\sum_{j=1}^{\text{N}_{\text{g}}} (j-\mu_{\text{y}})^{2}\sum_{i=1}^{Ng} P(i,j)}$$

$$p_{x}\left(i\right)=\sum_{j=1}^{N_{g}} P\left(i,j\right)$$

$$p_{y}\left(j\right)=\sum_{i=1}^{N_{g}} P\left(i,j\right)$$

$$p_{x+y}\left(k\right)=\sum_{i=1}^{N_{g}} \sum_{j=1}^{N_{g}} P\left(i,j\right), i+j=k,k=2,3,\ldots2N_{g}$$

$$p_{x-y}\left(k\right)=\sum_{i=1}^{N_{g}} \sum_{j=1}^{N_{g}} P\left(i,j\right), \left|i-j\right|=k,k=0,1,\ldots N_{g}-1$$

$$\mu_{x\text{-y}}\text{=}\sum_{k=0}^{\text{N}_{\text{g}}-1} kP_{\text{x-y}}(k)$$

$$HXY1=-\sum_{i=1}^{N_{g}} \sum_{j=1}^{N_{g}} P\left(i,j\right)\log_{2}\left(p_{x}\left(i\right)p_{y}\left(j\right)\right)$$

$$HXY2=-\sum_{i=1}^{N_{g}} \sum_{j=1}^{N_{g}} P_{x}\left(i\right)P_{y}\left(j\right)\log_{2}\left(p_{x}\left(i\right)p_{y}\left(j\right)\right)$$

$$HX=-\sum_{i=1}^{N_{g}} p_{x}(i)\log_{2}[p_{x}(i)]$$

$$HY=-\sum_{j=1}^{N_{g}} p_{y}(j)\log_{2}[p_{y}(j)]$$

$$H=-\sum_{i=1}^{N_{g}} \sum_{j=1}^{N_{g}} p(i,j)\log_{2}[p(i,j)]$$

The various radiomics features based on the co-occurrence matrix are then defined as:

1. Energy, called Uniformity in [2], also called Angular second moment in [3]:

$$energy=\sum_{i=1}^{N_{g}} \sum_{j=1}^{N_{g}} \left[P\left(i,j\right)\right]^{2}$$

1. Entropy:

$$entropy=-\sum_{i=1}^{N_{g}} \sum_{j=1}^{N_{g}} P\left(i,j\right)\log_{2}\left[P\left(i,j\right)\right]$$

1. Difference entropy (DiffEntropy):

$$difference entropy=-\sum_{k=0}^{N_{g}-1} P_{x-y}\left(k\right)\log_{2}\left[P_{x-y}\left(k\right)\right]$$

1. Sum entropy (SumEntropy):

$$sum entropy=-\sum_{k=2}^{2N_{g}} P_{x+y}\left(k\right)\log_{2}\left[P_{x+y}\left(k\right)\right]$$

1. Variance:

$$variance=\frac{1}{N_{g}\times N_{g}}\sum_{i=1}^{N_{g}} \sum_{j=1}^{N_{g}} [(i-\mu_{x})^{2}+(j-\mu_{y})^{2}]p(i,j)$$

1. Sum of squares variance (SumSquVar):

$$\text{s}um\text{ }of\text{ }squares\text{ }variance=\sum_{i=1}^{N_{g}} \sum_{j=1}^{N_{g}} \left(i-\mu\right)^{2}P\left(i,j\right)$$

1. Sum variance (SumVar):

$$sum variance=\sum_{k=2}^{2N_{g}} \left(k-SA\right)^{2}P_{x+y}\left(k\right)$$

where SA is Sum average2.

1. Maximum probability (MaxPossilility):

$$maximum probability=\max\left\{P\left(i,j\right)\right\}$$

1. Contrast:

$$contrast=\sum_{i=1}^{N_{g}} \sum_{j=1}^{N_{g}} \left|i-j\right|^{2}P\left(i,j\right)$$

1. Dissimilarity:

$$dissimilarity=\sum_{i=1}^{N_{g}} \sum_{j=1}^{N_{g}} \left|i-j\right|P\left(i,j\right)$$

1. Homogeneity, also called Inverse difference in [2]:

$$homogeneity =\sum_{i=1}^{N_{g}} \sum_{j=1}^{N_{g}} \frac{P\left(i,j\right)}{1+\left|i-j\right|}$$

1. Inverse Different Moment (InDiffMoment), also called local homogeneity in [4]:

$$inverse\text{ }different\text{ }moment=\sum_{i=1}^{N_{g}} \sum_{j=1}^{N_{g}} \frac{P\left(i,j\right)}{1+\left|i-j\right|^{2}}$$

1. Correlation:

$$correlation=\sum_{i=1}^{N_{g}} \sum_{j=1}^{N_{g}} \frac{(i-\mu_{x})(j-\mu_{y})p(i,j)}{\sigma_{x}\sigma_{y}}\text{=}\frac{\sum_{i=1}^{N_{g}} \sum_{j=1}^{N_{g}} ijP\left(i,j\right)-\mu_{x}\mu_{y}}{\sigma_{x}\sigma_{y}}$$

1. Difference Variance (DiffVar):

$$difference variance=-\sum_{k=0}^{N_{g}-1} \left(k-\mu_{x-y}\right)^{2}P_{x-y}\left(k\right)$$

$$\mu_{x\text{-y}}\text{=}\sum_{k=0}^{\text{N}_{\text{g}}-1} kP_{\text{x-y}}(k)$$

1. Auto correlation (AutoCorrelation):

$$auto correlation=\sum_{i=1}^{N_{g}} \sum_{j=1}^{N_{g}} ijp\left(i,j\right)$$

1. Cluster prominence (ClusterPro):

$$cluster prominence=\sum_{i=1}^{N_{g}} \sum_{j=1}^{N_{g}} \left[i+j-\mu_{x}-\mu_{y}\right]^{4}P\left(i,j\right)$$

1. Cluster shade (ClusterShade):

$$cluster shade=\sum_{i=1}^{N_{g}} \sum_{j=1}^{N_{g}} \left[i+j-\mu_{x}-\mu_{y}\right]^{3}P\left(i,j\right)$$

1. Cluster tendency (ClusterTen):

$$cluster tendency=\sum_{i=1}^{N_{g}} \sum_{j=1}^{N_{g}} \left[i+j-\mu_{x}-\mu_{y}\right]^{2}P\left(i,j\right)$$

1. Informational measure of correlation 1 (IMC1):

$$IMC1=\frac{H-HXY1}{\max\left\{HX,HY\right\}}$$

Where *HX and HY* are the entropies of $p_{x}(i)$ and $p_{y}(\text{j})$.

1. Informational measure of correlation 2 (IMC2):

$$IMC2=\sqrt{1-e^{-2\left(HXY2-H\right)}}$$

where *H* is the entropy of $p(i,j)$.

1. Inverse variance (InVar):

$$inverse variance=\sum_{i=1}^{N_{g}} \sum_{j=1}^{N_{g}} \frac{P\left(i,j\right)}{\left|i-j\right|^{2}},i\neq j$$

1. Inverse Difference Moment Normalized (IDMN):

$$IDMN=\sum_{i=1}^{N_{g}} \sum_{j=1}^{N_{g}} \frac{P\left(i,j\right)}{1+\left(\frac{\left|i-j\right|^{2}}{N^{2}}\right)}$$

1. Inverse Difference Normalized (IDN):

$$IDN=\sum_{i=1}^{N_{g}} \sum_{j=1}^{N_{g}} \frac{P\left(i,j\right)}{1+\left(\frac{\left|i-j\right|}{N}\right)}$$

1. Sum average1:

$$sum average1=\frac{1}{N_{g}\times N_{g}}\sum_{i=1}^{N_{g}} \sum_{j=1}^{N_{g}} [iP(i,j)+jP(i,j)]$$

1. Sum average2:

$$sum average2=\sum_{k=2}^{2N_{g}} \left[kP_{x+y}\left(k\right)\right]$$

1. Agreement:

$$agreement=\frac{P_{o}-P_{e}}{1-P_{e}}$$

where $P_{o}=\sum_{i=1}^{Ng} P(i,i)$ $P_{e}=\sum_{i=1}^{Ng} P(i,:)P(:,i)$

- ***Gray Level Run Length Matrix-based features (GLRLM)-13***

Gray level run length matrix-based features are described by Galloway et al. [5]. The element of GLRLM $P(i,j)$ counts the number of runs $j$ with collinearly adjacent pixels having the same gray level intensity $i$ as follows:

$$P(i,j)=\left\{\text{j | }I_{1}=\text{i},I_{2}=\text{i},...,I_{j}=\text{i}\right\}$$

where $I_{1},I_{2},...,I_{j}$ are collinearly adjacent voxels.

The GLRLM feature value was derived by considering all the 13 directions simultaneously, thus arriving at a single matrix. Let $P(i,j)$ be the $(i,j)\text{ }th$ entry in the given run-length matrix, $N_{g}$ the number of discrete intensity values in the image, $N_{r}$ the number of different run lengths, $N_{p}$ the number of voxels in the image, and the entry $(i,j)$ of the normalized GLRLM defined as:

$$p(i,j)=\frac{P(i,j)}{\sum_{i=1}^{N_{g}} \sum_{j=1}^{N_{r}} P(i,j)}$$

$$\mu_{i}=\sum_{i=1}^{N_{g}} i\sum_{j=1}^{N_{r}} p(i,j)$$

$$\mu_{j}=\sum_{j=1}^{N_{r}} j\sum_{i=1}^{N_{g}} p(i,j)$$

Then the GLRLM-based features are defined as:

1. Short Run Emphasis (SRE):

$$SRE=\sum_{i=1}^{N_{g}} \sum_{j=1}^{N_{r}} \left[\frac{p\left(i,j\right)}{j^{2}}\right]$$

1. Long Run Emphasis (LRE):

$$LRE=\sum_{i=1}^{N_{g}} \sum_{j=1}^{N_{r}} j^{2}p\left(i,j\right)$$

1. Gray Level Non-Uniformity (GLN):

$$GLN=\sum_{i=1}^{N_{g}} \left[\sum_{j=1}^{N_{r}} p\left(i,j\right)\right]^{2}$$

1. Run Length Non-Uniformity (RLN):

$$RLN=\sum_{j=1}^{N_{r}} \left[\sum_{i=1}^{N_{g}} p\left(i,j\right)\right]^{2}$$

1. Run Percentage (RP):

$$RP=\sum_{i=1}^{N_{g}} \sum_{j=1}^{N_{r}} \frac{p\left(i,j\right)}{N_{p}}$$

1. Low Gray Level Run Emphasis (LGRE):

$$LGRE=\sum_{i=1}^{N_{g}} \sum_{j=1}^{N_{r}} \left[\frac{p\left(i,j\right)}{i^{2}}\right]$$

1. High Gray Level Run Emphasis (HGRE):

$$HGRE=\sum_{i=1}^{N_{g}} \sum_{j=1}^{N_{r}} i^{2}p\left(i,j\right)$$

1. Short Run Low Gray Level Emphasis (SRLGE):

$$SRLGE=\sum_{i=1}^{N_{g}} \sum_{j=1}^{N_{r}} \left[\frac{p\left(i,j\right)}{i^{2}j^{2}}\right]$$

1. Short Run High Gray Level Emphasis (SRHGE):

$$SRHGE=\sum_{i=1}^{N_{g}} \sum_{j=1}^{N_{r}} \left[\frac{p\left(i,j\right)i^{2}}{j^{2}}\right]$$

1. Long Run Low Gray Level Emphasis (LRLGE):

$$LRLGE=\sum_{i=1}^{N_{g}} \sum_{j=1}^{N_{r}} \left[\frac{p\left(i,j\right)j^{2}}{i^{2}}\right]$$

1. Long Run High Gray Level Emphasis (LRHGE):

$$LRHGE=\sum_{i=1}^{N_{g}} \sum_{j=1}^{N_{r}} p\left(i,j\right)i^{2}j^{2}$$

1. Gray Level Variance (GLV)

$$GLV=\frac{1}{N_{g}\times N_{r}}\sum_{i=1}^{N_{g}} \sum_{j=1}^{N_{r}} (ip(i,j)-\mu_{i})^{2}$$

1. Run length Variance (RLV)

$$RLV=\frac{1}{N_{g}\times N_{r}}\sum_{i=1}^{N_{g}} \sum_{j=1}^{N_{r}} (jp(i,j)-\mu_{j})^{2}$$

- ***Gray Level Size Zone Matrix-based features (GLSZM)-13***

Gray-level size-zone matrix-based features, was described in [1]. GLSZM describes the number of a certain size zone $j$ having same intensity $i$ within N-connected neighbors in a 3D space as follows:

$$P(i,j)=\left\{\text{j | }I_{1}=\text{i},I_{2}=\text{i},...,I_{j}=\text{i}\right\}$$

where voxels $I_{1},I_{2},...,I_{j}$ are within N-connected neighbors (N=26).

Let $P(i,j)$ be the $(i,j)th$ entry in the given size-zone matrix, $N_{g}$ the number of discrete intensity values in the image, $N_{z}$ the size of the largest homogeneous region in the volume of interest, and $N_{\alpha}$ the number homogeneous zones in the image. The entry $(i,j)$ of the GLSZM are then normalized as:

$$p(i,j)=\frac{P(i,j)}{\sum_{i=1}^{N_{g}} \sum_{j=1}^{N_{z}} P(i,j)}$$

$$\mu_{i}=\sum_{i=1}^{N_{g}} i\sum_{j=1}^{N_{z}} p(i,j)$$

$$\mu_{j}=\sum_{j=1}^{N_{z}} j\sum_{i=1}^{N_{g}} p(i,j)$$

The GLSZM-based features are then defined as:

1. Small Zone Emphasis (SZE):

$$SZE=\sum_{i=1}^{N_{g}} \sum_{j=1}^{N_{z}} \left[\frac{p\left(i,j\right)}{j^{2}}\right]$$

1. Large Zone Emphasis (LZE):

$$LZE=\sum_{i=1}^{N_{g}} \sum_{j=1}^{N_{z}} j^{2}p\left(i,j\right)$$

1. Gray Level Non-uniformity (GLN) also called Intensity Variability (IV) in [6]:

$$GLN=\sum_{i=1}^{N_{g}} \left[\sum_{j=1}^{N_{z}} p\left(i,j\right)\right]^{2}$$

1. Zone Size Non-uniformity (ZSN) also called Size Zone Variability (SZV) in [6]:

$$ZSN=\sum_{j=1}^{N_{z}} \left[\sum_{i=1}^{N_{g}} p\left(i,j\right)\right]^{2}$$

1. Zone Percentage (ZP):

$$ZP=\sum_{i=1}^{N_{g}} \sum_{j=1}^{N_{z}} \frac{p\left(i,j\right)}{N_{\alpha}}$$

1. Low Gray Level Zone Emphasis (LGZE) also called Low Intensity Emphasis (LIE) in [6]:

$$LGZE=\sum_{i=1}^{N_{g}} \sum_{j=1}^{N_{z}} \left[\frac{p\left(i,j\right)}{i^{2}}\right]$$

1. High Gray level Zone Emphasis (HGZE) also called High Intensity Emphasis (HIE) in [6]:

$$HGZE=\sum_{i=1}^{N_{g}} \sum_{j=1}^{N_{z}} i^{2}p\left(i,j\right)$$

1. Small Zone Low Gray Level Emphasis (SZLGE) also called Low Intensity Small Area Emphasis (LISAE) in [6]:

$$SZLGE=\sum_{i=1}^{N_{g}} \sum_{j=1}^{N_{z}} \left[\frac{p\left(i,j\right)}{i^{2}j^{2}}\right]$$

1. Small Zone High Gray-Level Emphasis (SZHGE) also called High Intensity Small Area Emphasis (HISAE) in [6]:

$$SZHGE=\sum_{i=1}^{N_{g}} \sum_{j=1}^{N_{z}} \left[\frac{p\left(i,j\right)i^{2}}{j^{2}}\right]$$

1. Large Zone Low Gray-Level Emphasis (LZLGE) also called Low Intensity Large Area Emphasis (LILAE) in [6]:

$$LZLGE=\sum_{i=1}^{N_{g}} \sum_{j=1}^{N_{z}} \left[\frac{p\left(i,j\right)j^{2}}{i^{2}}\right]$$

1. Large Zone High Gray-Level Emphasis (LZHGE) also called High Intensity Large Area Emphasis (HILAE) in [6]:

$$LZHGE=\sum_{i=1}^{N_{g}} \sum_{j=1}^{N_{z}} p\left(i,j\right)i^{2}j^{2}$$

1. Gray Level Variance (GLV)

$$GLV=\frac{1}{N_{g}\times N_{z}}\sum_{i=1}^{N_{g}} \sum_{j=1}^{N_{z}} (ip(i,j)-\mu_{i})^{2}$$

1. Zone Size Variance (ZSV)

$$ZSV=\frac{1}{N_{g}\times N_{z}}\sum_{i=1}^{N_{g}} \sum_{j=1}^{N_{z}} (jp(i,j)-\mu_{j})^{2}$$

where zone aforesaid also called area in [6].

- ***Neighborhood Gray Tone Difference Matrix–based features (NGTDM)-5***

NGTDM is a column matrix [7]. Denote the$i^{th}$entry of the NGTDM as$P(i)$, defined as:

$$P(i)=\left\{\begin{matrix} \sum_{i\in\left\{\left.N_{i}\right\}\right.} \left|i-\overline{A_{i}}\right|\text{ }if\text{ }N_{i}\text{>0,} \\ 0\text{ }otherwise. \end{matrix}\right.$$

where $\{N_{i}\}$ is the set of all voxels with gray-level $i$ in tumor volume (including the peripheral region), $N_{i}$ is the number of voxels with gray-level $i$ in tumor volume, and $A_{i}$ is the average gray level of the $M$ connected neighbors around a center voxel $V(i,j,k)$ with gray level $i$. Also, we have

$$\overline{A_{i}}=\overline{A(}j,k,l)=\frac{1}{M}\sum_{m=-d}^{d} \sum_{n=-d}^{d} \sum_{s=-d}^{d} V(j+m,k+n,l+s),(m,n,l)\neq(0,0,0)$$

where $d=1$, specifies the window size as $3\times3\times3$, and $M=(2d+1)^{3}-1$. The quantity $n_{i}=\frac{N_{i}}{N}$ is also defined, where $N$ is the total number of voxels in tumor volume. The NGTDM-based features are then defined as:

1. Coarseness:

$$coarseness=[\varepsilon+\sum_{i=1}^{N_{g}} n_{i}P(i)]^{-1}$$

where $\varepsilon$ is a small number to prevent coarseness becoming infinite, *N_g_* the number of discrete intensity values in the image.

1. Contrast:

$$contrast=[\frac{1}{N_{g}\times(N_{g}-1)}\sum_{i=1}^{N_{g}} \sum_{j=1}^{N_{g}} n_{i}n_{j}(i-j)^{2}]\times[\frac{1}{N}\sum_{i=1}^{N_{g}} P(i)]$$

1. Busyness:

$$busyness=\frac{\sum_{i=1}^{N_{g}} n_{i}P(i)}{\sum_{i=1}^{N_{g}} \sum_{j=1}^{N_{g}} \left|in_{i}-jn_{j}\right|},n_{i}\neq0,n_{j}\neq0$$

1. Complexity:

$$complexity=\sum_{i=1}^{N_{g}} \sum_{j=1}^{N_{g}} \frac{\left|i-j\right|\left[n_{i}P(i)+n_{j}P(j)\right]}{N(n_{i}+n_{j})},n_{i}\neq0,n_{j}\neq0$$

1. Strength:

$$strength=\frac{\sum_{i=1}^{N_{g}} \sum_{j=1}^{N_{g}} (n_{i}\text{+}n_{j})(i-j)^{2}}{\varepsilon+\sum_{i=1}^{N_{g}} P(i)},n_{i}\neq0,n_{j}\neq0$$

where $\varepsilon$ is a small number to prevent strength becoming infinite.

**References**

1. Thibault G et al (2009) Texture indexes and gray level size zone matrix application to cell nuclei classification. Pattern Recognition Inf Process 140-150
2. Gomez W, Pereira W C and Infantosi A F (2012) Analysis of co-occurrence texture statistics as a function of gray-level quantization for classifying breast ultrasound. IEEE Trans Med Imaging 31: 1889-1899
3. Lee J et al (2015) Texture feature ratios from relative CBV maps of perfusion MRI are associated with patient survival in glioblastoma. AJNR Am J Neuroradiol 37:37-43
4. El N I et al (2009) Exploring feature-based approaches in PET images for predicting cancer treatment outcomes. Pattern Recognit 42: 1162-1171
5. Galloway M M (1974) Texture analysis using grey level run lengths. NASA STI/Recon Technical Report N. 75: 18555.
6. Leijenaar R T et al. (2013) Stability of FDG-PET Radiomics features: An integrated analysis of test-retest and inter-observer variability. Acta Oncol 52: 1391-1397.
7. Amadasun M and King R (1989) Textural features corresponding to textural properties. IEEE Trans Sys Man Cyb 19: 1264-1274.
